# Mesenchymal stem cells for treating autoimmune dacryoadenitis

**DOI:** 10.1186/s13287-017-0593-3

**Published:** 2017-06-05

**Authors:** Xiaoxiao Lu, Xilian Wang, Hong Nian, Dan Yang, Ruihua Wei

**Affiliations:** 1 Tianjin Medical University Eye Hospital, Tianjin Medical University Eye Institute & Tianjin Medical University School of Optometry and Ophthalmology, No.251 Fukang Road, Nankai District, Tianjin, 300384 People’s Republic of China; 2 Tianjin Beichen Hospital, No. 7, Beiyi Road, Beichen District, Tianjin, 300400 China

**Keywords:** Mesenchymal stem cells, Autoimmune dacryoadenitis, Immunomodulation

## Abstract

Autoimmune dacryoadenitis, such as Sjögren syndrome, comprises multifactorial and complex diseases. Inflammation of the lacrimal gland plays a key role in the pathogenesis of diseases. Unfortunately, current treatment strategies, including artificial tears, anti-inflammatory drugs, punctual occlusion, and immunosuppressive drugs, are only palliative, and long-term administration of these strategies is associated with adverse effects that limit their utility. Hence, an effective and safe treatment for autoimmune dacryoadenitis is urgently needed. Mesenchymal stem cells (MSCs) have emerged as a promising tool for treating autoimmune dacryoadenitis, owing to their immunosuppressive properties, tissue repair functions, and powerful differentiation capabilities. A large number of studies have focused on the effect of MSCs on autoimmune diseases, such as autoimmune uveitis, inflammatory bowel disease, and collagen-induced arthritis, but few studies have, to date, unequivocally established the efficacy of MSCs for treating autoimmune dacryoadenitis. In this review, we discuss recent advances in MSC treatment for autoimmune dacryoadenitis.

## Background

Autoimmune dacryoadenitis, such as Sjögren syndrome (SS), comprises multifactorial and complex diseases characterized by lymphocyte infiltration of lacrimal glands, which induces functional and occupational disability [1]. The prevalence of autoimmune dacryoadenitis in people is dramatically increasing. Current treatment strategies include artificial tears, nonsteroidal or corticosteroidal anti-inflammatory agents, immunosuppressive drugs and punctual occlusion [2]. However, these therapeutic modalities provide just palliative relief and are associated with adverse effects. An accumulating body of evidence supports the notion that the pathogenesis of these diseases results from immune disorders caused by the imbalance of Th1/Th17 cells and aberrant T regulatory cells (Tregs) [3, 4]. Accordingly, therapies that regulate or inhibit immune responses may be useful. One method to modulate immune responses is the administration of mesenchymal stem cells (MSCs).

MSCs are multipotent nonhematopoietic stem cells which have the ability to differentiate into a variety of cell types, such as adipocytes, osteocytes, fibroblasts, and smooth muscle cells; under special conditions, they even differentiate into epithelial, endothelial, cardiac, and stromal cells [5, 6]. Along with their ability to differentiate into cells of multiple lineages, MSCs have attracted great interest because of their immunosuppressive properties, tissue repair functions, and powerful differentiation capabilities. The application of MSCs in autoimmune diseases has been summarized in greater detail in a review by Sánchez-Berná I [7], although this covered treatments for a variety of immune diseases. In this review, we summarize the immunomodulation and treatment effects of MSCs.

### Immunoregulatory mechanisms and potential of MSCs

#### Immunomodulatory and anti-inflammation potential

MSCs exert immunoregulatory and anti-inflammation potential through cell-to-cell contact and the release of cytokines [8]. They may modulate T and B lymphocytes, natural killer cells (NK cells), and antigen-presenting cells (APCs) such as dendritic cells, which makes them a promising therapy for autoimmune disease [9]. The regulation of immune cells by MSCs mainly relies on a panel of cytokines secreted by the MSCs, including IL-10, transforming growth factor-β (TGF-β), prostaglandin E2 (PGE2), indoleamine-2,3-dioxygenase (IDO), human leukocyte antigen-G5 (HLAG), and nitric oxide [10, 11]. MSCs may play a strong immunosuppressive role by secreting various cytokines such as intercellular cell adhesion molecule-1 (ICAM-1), CXC chemokine ligand-10 (CXCL-10), C-C motif chemokine-8 (CCL-8), and IDO in inflammatory conditions [10]. Moreover, they may prevent the expression of the cytokines interferon-γ (IFN-γ) and tumor necrosis factor-α (TNF-α) by Th1 cells and increase the level of Th2-related cytokines, including IL-10 and IL-4 [11]. In addition, MSCs facilitate the formation of the immune microenvironment via promoting the proliferation and activation of Tregs [12].

#### Tissue repair function of MSCs

MSCs also have important potential in tissue repair function, which is an important characteristic for cell therapy [13]. When an organism is injured, MSCs may migrate to the injury site and differentiate into osteoblasts, fibroblasts, and other cells participating in the repair of the injured tissue under the regulation of the local environment [14]. At the same time, MSCs may also produce and secrete a variety of cytokines [15], including vascular endothelial growth factor (VEGF), fibroblast growth factor 2 (FGF-2), insulin-like growth factor 1 (IGF-1), and hepatocyte growth factor (HGF) [16]. These properties of MSCs together promote tissue repair.

The ability of MSCs to migrate to the site of injury and damaged tissue is called the homing ability. To date, the molecular mechanism of the homing ability is unclear, but inflammatory chemokines at the injury site play an important role in their migration. A large number of chemokine receptors are secreted by MSCs and are used in conjunction with chemokines released by the injured tissue. Chemokine activation may cause the MSCs to migrate to the injury site and exert their tissue repair function [17]. The axis of stromal cell-derived factor 1α (SDF-1α) and its receptor CXCR4 is an important biological axis which promotes MSC homing to damaged tissue [18]. Some studies report that CCL21 and its specific receptor, CCR7, as well as some vascular cell adhesion factors, matrix metalloproteinases (MMPs) and integrins, also participate in the homing of MSCs [19].

#### Low immunogenicity and genetic modification of MSCs

Based on previous research, MSCs possess hypoimmunogenic properties in vitro and in vivo, so-called immune privilege. An accumulating body of evidence supports the notion that the mechanisms of immune privilege are most likely due to low expression levels or a lack of surface expression of class I major histocompatibility complex (MHC-I), MHC-II, Fas ligand (FasL), and co-stimulatory molecules (CD40, CD86, and CD80), which are required for activating lymphoid cells, including T cells, B cells, NK cells, and dendritic cells [11]. The low expression levels of MHC-I could be advantageous for MSCs to escape immune clearance [11]. A lack of surface expression of MHC-II could protect MSCs from CD4^+^ T-cell recognition. MSCs were not recognized and induced to apoptosis by Fas-expressing immunocytes because of a lack of expression of FasL. In addition, MSCs do not express T-cell co-stimulatory molecules which are needed for the activation of T cells [20]. Based on these properties, MSCs do not promote a proliferative T-cell response and challenge the response of allogeneic immune cells. However, some studies have differed, presuming that the MHC levels expressed by MSCs were affected by proinflammatory cytokines, such as IFN-γ [21, 22]. MSCs may express higher levels of MHC under the condition of low expression of IFN-γ, while MHC expression levels were low during overexpression of IFN-γ [21].

Because of their unique advantages—the easy with which they can be genetically modified and their low immunogenicity—MSCs have gradually become a mainstay of gene therapy. MSCs, as a vector for gene therapy, may endogenously express genes efficiently and stably, avoiding the short biological half-life of exogenously expressed genes in vivo and the production of antibodies owing to multiple injections [23]. Genetically modified MSCs have been used in various diseases. MSCs which have been modified by incorporating anti-inflammatory factor or cell growth factor genes not only maintain the original characteristics of the cells but also have the advantage of producing anti-inflammatory factors [24] (such as IL-10 and TGF-β) or growth factors, including HGF and basic FGF (bFGF). Genetically modified MSCs may maximize the benefit of cell therapy and gene therapy [25].

### MSCs in autoimmune dacryoadenitis

#### Experimental autoimmune dacryoadenitis

The immunoregulation and treatment effect of MSCs was first studied in experimental autoimmune encephalomyelitis [26], where multiple sclerosis symptoms were alleviated after the administration of MSCs in mice. Similar anti-inflammatory effects of MSCs have also been demonstrated in other autoimmune diseases, such as rheumatoid arthritis [27] and graft versus host disease (GVHD) [28]. Khalili et al. [29] studied the immunomodulatory and protective effects of MSCs in the salivary gland, suggesting that MSCs exert their treatment effect through tissue repair, anti-inflammation, and immunoregulation [30]. These findings have potential implications for future investigations.

The dog is considered to be a superior animal model of autoimmune dacryoadenitis because dogs develop the disease naturally [31] and they have great similarities with human beings [32]. Park et al. [33] validated that MSCs are safe, with no changes in appetite and fecal output noted in dogs treated with adipose-derived MSCs.

To study the immunomodulatory effects of MSCs, Villatoro et al. [34] implanted MSCs topically into the lacrimal gland and the gland of the third eyelids in 12 dogs affected by bilateral dry eye disease and refractory to conventional treatments. After 9 months’ follow-up, researchers supposed that the possible mechanism of the treatment effect of MSCs in dry eye disease is based on their anti-inflammatory effect, stimulated by released proinflammatory cytokines (TNF, IFN, IL-6) [35] and through secretion of immunomodulatory soluble factors such as TGF-β, PGE2, HGF, and IDO [36]. Lee et al. [37] reported that tear production was increased significantly, while the cytokines IL-2 and IFN-γ secreted by T lymphocytes were decreased and the infiltration of CD3+ or CD4+ cells was alleviated after the injection of MSCs. These results provide evidence that MSCs exert their immunomodulatory effects by inhibiting the infiltration of CD3+ and CD4+ cells, suppressing the proliferation and differentiation of T lymphocytes [38]. In addition, Yao et al. [39] discovered that MSCs promote the secretion of anti-inflammatory factors (TGF-β) and antiangiogenic agents (thrombospondin-1) and decreased the level of the inflammatory factor TNF-α, chemokines (MIP-1α, MCP-1), and the angiogenesis factor VEGF.

In addition, MSCs are known to promote tear production [40] and tissue repair, increase the density of corneal epithelial cells, and protect conjunctival goblet cells from damage. MSCs reduced the expression of inflammatory factors such as MMP2 and IL-2 and reconstruct severely damaged rat corneal surface after the transplantation of human MSCs on amniotic membrane [41]. Beyazyildiz et al. [2] reported that increased numbers of goblet cells were present in conjunctiva in MSC-treated rats and these cells contained more secretory granules in their cytoplasm. Moreover, microvilli were preserved at apical portions of the corneal epithelium of MSC-treated rats and there was no prominent sign of cellular injury in the cornea of these rats [2]. Similar reports have suggested that MSCs increased goblet cell counts and restored goblet cells in the conjunctiva [37].

#### Clinical study of autoimmune dacryoadenitis

Autoimmune dacryoadenitis is a common complication in patients with GVHD or SS [42]. Given the immunomodulatory and anti-inflammatory effects of MSCs, some researchers have studied the treatment effects of MSCs in patients with Sjögren syndrome or chronic GVHD (cGVHD).

Human SS is a chronic, systemic autoimmune disorder characterized by inflammation of the exocrine glands and functional impairment of the salivary and lacrimal glands [43]. Xu et al. [44] performed a clinical evaluation of allogeneic MSC treatment on patients with primary SS who were poorly responsive to conventional therapies. All patients tolerated allogeneic MSCs well and showed improvements in symptoms during or after MSC infusion, and no adverse events occurred. Further experiments revealed that MSC treatment completely abolished production of anti-SSA/Ro and downregulated anti-SSB/La levels in serum. Data suggested that MSCs may exert their potent curative effects by suppressing T follicular helper (Tfh) cell differentiation and function. Together, the results demonstrated that MSC treatment substantially ameliorated disease symptoms, increased salivary flow rate, and inhibited inflammatory responses [44]. Subsequent experiments showed that CD4+ T cells secreted some soluble factors to contribute to IDO secretion by MSCs, and the secretion of IDO may play a role in the inhibitory effect of MSCs on the differentiation of circulating Tfh cells [45].

cGVHD is a serious common long-term complication of allogeneic hematopoietic stem cell treatment [46]. Ocular surface damage is one of the most common pathological manifestations in patients with cGVHD and occurs in up to 80% of patients [42, 47, 48]. The symptoms of dry eye patients secondary to cGVHD were alleviated by infusion of MSCs, even though little is known about the mechanisms used by MSCs to suppress the symptoms of dry eye associated with cGVHD. Researchers have reported that MSCs exerted their potent curative effects by suppressing both local and systemic inflammation, such as inhibiting the activation and proliferation of T cells [49]. Also, MSCs increased lacrimal secretions and improved symptoms by alleviating the inflammation and fibrosis of lacrimal glands [50]. Recent studies have increasingly recognized the important effects of MSCs for restoring Th1/Th2 homeostasis, demonstrating that the efficiency of MSC treatment depends on an increase in CD8+ CD28− T cells [38].

## Conclusions

**Fig. 1:**
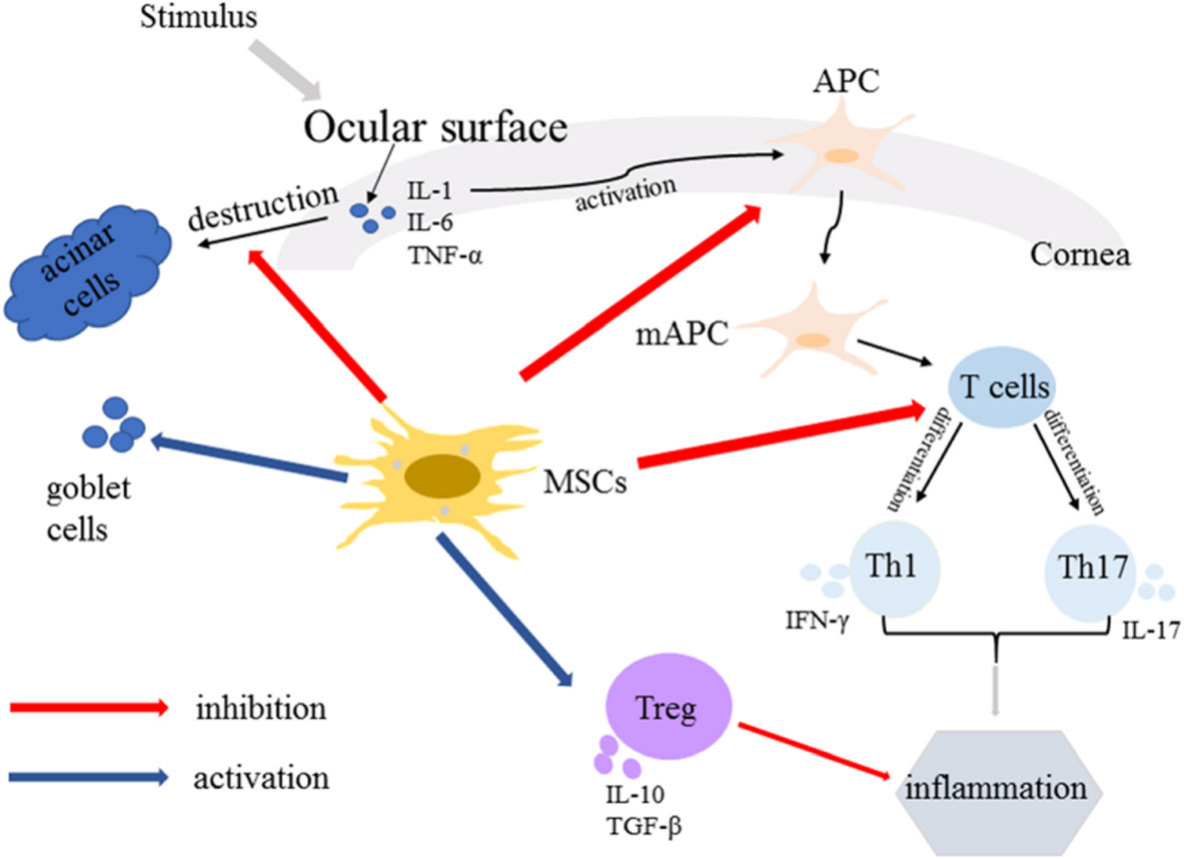
The mechanisms of autoimmune dacryoadenitis and MSC treatment for it. Both the innate and adaptive immune systems participate in the pathogenesis of autoimmune dacryoadenitis, which occurs when the ocular surface is stimulated by various factors. Cytokines such as IL-6, IL-1, and TNF-α have been suggested to play a role in the destruction of acinar cells. The proinflammatory milieu promotes the activation and maturation of APCs, which contribute to the induction of Th1 cells and Th17 cells. IFN-γ and IL-17, separately secreted by Th1 and Th17 cells, promote the production of proinflammatory cytokines/chemokines which induce ocular surface inflammation. MSCs protect the ocular surface by inhibiting the infiltration of inflammatory cells, restoring Th1/Th2 homeostasis, suppressing Th17 cells, activating Tregs, stimulating epithelial cells, and promoting goblet cell regeneration. They inhibit the activation and proliferation of T cells and favor the differentiation of T cells into Th2 cells, thus restoring Th1/Th2 homeostasis. Furthermore, MSCs could exert their protective effects by activating Tregs and suppressing Th17 cells. In addition, MSCs promote tissue repair by stimulating epithelial cell and goblet cell regeneration

Autoimmune dacryoadenitis, a persistent and difficult to cure ocular surface disease, affects patients’ quality of life seriously and even induces severe visual impairment. It is a multifactorial and complex disease and inflammation plays a key role in its pathogenesis [2] (Fig. 1). MSCs have shown therapeutic effect in autoimmune dacryoadenitis through several mechanisms: inhibition of inflammatory cell infiltration and inflammatory cytokine release, restoration of Th1/Th2 homeostasis, activation of Tregs, alleviation of fibrosis of lacrimal glands, and stimulation of epithelial cell regeneration (Fig. 1). Further studies should focus on the exact mechanism of MSCs, their long-term effects on clinical outcomes and safety, the duration of therapy, and the optimal timing and dosages for treating autoimmune dacryoadenitis.

## Abbreviations

APC: Antigen-presenting cell
cGVHD: Chronic graft-versus-host disease
FasL: Fas ligand
FGF: Fibroblast growth factor
GVHD: Graft-versus-host disease
HGF: Hepatocyte growth factor
IDO: Indoleamine-2,3-dioxygenase
IFN-γ: Interferon-γ
IGF-1: Insulin-like growth factor 1
IL: Interleukin
MHC: Major histocompatibility complex
MMP: Matrix metalloproteinase
MSC: Mesenchymal stem/stromal cell
PGE2: Prostaglandin E2
SS: Sjögren syndrome
Tfh cells: T follicular helper cell
TGF-β: Transforming growth factor-β
TNF-α: Tumor necrosis factor-α
Treg: T regulatory lymphocyte
VEGF: Vascular endothelial growth factor
